# Supplementary material for: Recent genome reduction of Wolbachia in Drosophila recens targets phage WO and narrows candidates for reproductive parasitism
Source: PeerJ. 2014 Aug 14;2:e529. doi: 10.7717/peerj.529 (PMC4137656; doi:10.7717/peerj.529)
Supplement: Table S1 — Missing genes are defined as any wMel genes without BLASTn hits to assembled wRec scaffolds (E-value < 10−10) and also absent when raw wRec/host sequencing reads were mapped to these genes as reference templates. [file peerj-02-529-s001.doc]

| *Locus Tag* | *Gene* |
| --- | --- |
|  |  |
| *Non-phage* |  |
| WD_0032 | Hypothetical Protein |
|  |  |
| *WO-A* |  |
| WD_0254 | Transcriptional Regulator, Putative |
| WD_0256 | Hypothetical Protein |
| WD_0257 | DUF2466, Truncation |
| WD_0259 | Conserved Hypothetical Protein |
| WD_0261 | Conserved Hypothetical Protein, Interruption-N |
| WD_0262 | Conserved Hypothetical Protein, Interruption-C |
| WD_0263 | Prophage LambdaW1, DNA Methylase |
| WD_0264 | Conserved Hypothetical Protein |
| WD_0265 | Prophage LambdaW1, Terminase Large Subunit, Putative |
| WD_0266 | gpW |
| WD_0267 | Hypothetical Protein |
| WD_0273 | Conserved Hypothetical Protein |
|  |  |
| *Octomom* |  |
| WD_0512 | Hypothetical Protein |
| WD_0513 | Hypothetical Protein |
| WD_0514 | Ankyrin Repeat Domain Protein |
|  |  |
| *WO-B* |  |
| WD_0564 | Hypothetical Protein |
| WD_0565 | Patatin Family Protein |
| WD_0566 | Ankyrin Repeat Domain Protein |
| WD_0567 | Prophage P2W3, Tail Protein D, Putative |
| WD_0568 | Prophage P2W3, Tail Protein X, Putative |
| WD_0569 | Prophage P2W3, Tail Protein U, Putative |
| WD_0570 | Prophage P2W3, Tail Tape Measure Protein, Truncated |
| WD_0571 | Prophage P2W3, Tail Tape Measure Protein, Truncated |
| WD_0572 | Tail Chaperone G/GT |
| WD_0573 | RelE Pseudogene |
| WD_0574 | Prophage P2W3, Contractile Tail Tube Protein |
| WD_0576 | Hypothetical Protein |
| WD_0577 | Hypothetical Protein |
| WD_0578 | Hypothetical Protein |
| WD_0579 | Hypothetical Protein |
| WD_0580 | Hypothetical Protein |
| WD_0581 | Hypothetical Protein |
| WD_0582 | Regulatory Protein RepA, Putative |
| WD_0583 | Conserved Hypothetical Protein, AAA_25 |
| WD_0584 | Hypothetical Protein |
| WD_0585 | Conserved Hypothetical Protein |
| WD_0586 | Hypothetical Protein |
| WD_0589 | Conserved Hypothetical Protein, AAA_25 |
| WD_0590 | Conserved Hypothetical Protein, AAA_24 |
| WD_0591 | Sigma 70 |
| WD_0594 | Prophage LambdaW4, DNA Methylase |
| WD_0595 | Conserved Hypothetical Protein |
